# Social Care Recommendations in National Diabetes Treatment Guidelines

**DOI:** 10.1007/s11892-022-01490-z

**Published:** 2022-08-30

**Authors:** Benjamin Aceves, Rose Gunn, Maura Pisciotta, Na’amah Razon, Erika Cottrell, Danielle Hessler, Rachel Gold, Laura M. Gottlieb

**Affiliations:** 1 Social Interventions Research and Evaluation Network, University of California San Francisco, San Francisco, CA USA; 2 School of Public Health, San Diego State University, San Diego, CA USA; 3 OCHIN, Inc., Portland, OR USA; 4 Department of Family and Community Medicine, University of California Davis, Sacramento, CA USA; 5 Kaiser Permanente Center for Health Research, Portland, OR USA

**Keywords:** Diabetes, Social care, Treatment guidelines, Health disparities

## Abstract

**Purpose of Review:**

An expanding body of research documents associations between socioeconomic circumstances and health outcomes, which has led health care institutions to invest in new activities to identify and address patients’ social circumstances in the context of care delivery. Despite growing national investment in these “social care” initiatives, the extent to which social care activities are routinely incorporated into care for patients with type II diabetes mellitus (T2D), specifically, is unknown. We conducted a scoping review of existing T2D treatment and management guidelines to explore whether and how these guidelines incorporate recommendations that reflect social care practice categories.

**Recent Findings:**

We applied search terms to locate all T2D treatment and management guidelines for adults published in the US from 1977 to 2021. The search captured 158 national guidelines. We subsequently applied the National Academies of Science, Engineering, and Medicine framework to search each guideline for recommendations related to five social care activities: Awareness, Adjustment, Assistance, Advocacy, and Alignment. The majority of guidelines (122; 77%) did not recommend any social care activities. The remainder (36; 23%) referred to one or more social care activities. In the guidelines that referred to at least one type of social care activity, adjustments to medical treatment based on social risk were most common [34/36 (94%)].

**Summary:**

Recommended adjustments included decreasing medication costs to accommodate financial strain, changing literacy level or language of handouts, and providing virtual visits to accommodate transportation insecurity. Ensuring that practice guidelines more consistently reflect social care best practices may improve outcomes for patients living with T2D.

## Introduction

Type II diabetes mellitus (T2D) affects one in every 10 people in the USA and is associated with a wide range of complications, including neuropathy, kidney disease, and cardiovascular disease [1, 2]. The disease and its comorbidities contribute to annual costs exceeding $300 billion, with over $200 billion in direct health care expenditures [3]. Despite substantial national investments in T2D prevention and treatment, the T2D age-adjusted death rate has increased nearly every year in the past decade [4, 5]. These rates have increased more rapidly in low-income and racialized populations—demonstrating pervasive health inequities [6•, 7]. The observed racial and ethnic disparities stem in part from the intertwining of race and poverty in the USA: financial insecurity, lack of transportation access, and low levels of literacy disproportionately affect US people of color [6•, 7], and these factors influence patients’ level of engagement in care [8, 9]. A new wave of innovations is needed to confront the US T2D epidemic and the deeply entrenched social and economic inequities that have exacerbated it.

To advance work on health inequity, numerous US health professional organizations have called for systematically integrating social care into health care delivery [10]. These calls have focused on both expanding social risk screening and increasing navigation supports to help patients obtain relevant social services [10]. Reflecting this growing interest in social care, agencies such as the Centers for Medicare and Medicaid Services (CMS) and the National Commission on Quality Assurance are currently considering performance measures related to social care screening and interventions under select programs. In parallel, numerous states are incorporating strategies to incentivize social care initiatives. The evidence base on both social risk screening and related interventions is also rapidly expanding [11].

In this paper, we examine how and to what extent national T2D treatment and management guidelines, specifically, have incorporated social care recommendations. Disease treatment and management guidelines are an important national resource for T2D care standardization; they provide clinicians, patients, payers, and other health care stakeholders specific evidence-based recommendations. To assess how T2D guidelines address social care, the research team relied on a framework described in a 2019 National Academies of Science, Engineering, and Medicine (NASEM) report that was the first national effort to articulate different types of medical and social care integration strategies [12]. The report delineates for five categories for social care activities at both the patient-, healthcare-, and community-levels: Awareness, Adjustment, Assistance, Alignment, and Advocacy (see Table 1). Though not specific to T2D, the report’s social care framework provides a scaffold for designing, identifying, and strengthening health care engagement in this rapidly expanding area of health care services.

**Table 1:** NASEM social care activities

Social care activity	Definition
Awareness	Recommendations for health care teams to collect information about patients’ social risks and assets (e.g., to conduct social risk screening
Adjustment	Recommendations for health care teams to adapt clinical care to mitigate impacts of patients’ social barriers on disease prevention or disease management
Assistance	Recommendations for health care teams to intervene on social risks by providing social services or referring patients to social services
Alignment	Recommendations for health care stakeholders to design internal practices and investments to complement community-level socioeconomic development initiatives
Advocacy	Recommendations to support and promote local, regional, and federal policies intended to improve community-level socioeconomic development

Using the NASEM framework, the analyses presented here examined if, how, and in what circumstances current T2D care guidelines incorporate social care activities (i.e., screening, intervention, and advocacy). This has important implications for social and T2D care integration: if the guidelines already include social care recommendations, the next step would be to assess the prevalence, facilitators, and barriers to providing such care; if they do not, future efforts should focus on strengthening health care services research on social care integration to ensure they are translatable to clinical treatment and management recommendations.

## Methods

Following the Preferred Reporting Items for Systematic Reviews and Meta-analysis (PRIMSA) recommendations, a systematic scoping review was conducted on T2D treatment and management guidelines [13]. Per methods applied in previous reviews, treatment and management guidelines were defined as published recommendations on T2D management sanctioned by a national health or health care professional organization [14, 15].

### Data Extraction

First, a medical librarian helped the research team develop a PubMed search strategy. All practice, treatment, management, and clinical guidelines were searched in Ovid Medline using the search term “diabetes.” The search was limited to guidelines published in the USA, written in English, and published before January 2022. Following the initial PubMed guideline search, research team members independently searched for additional guidelines from national T2D or endocrine professional organizations (e.g. American Diabetes Association (ADA), the American Association of Clinical Endocrinology (AACE), the National Institute of Diabetes and Digestive and Kidney Diseases (NIDDK), the National Diabetes Education Program (NDEP), and the American Association of Diabetes Educators (ADCES)) to ensure a comprehensive search.

Second, publication titles and abstracts from the initial guideline search were reviewed to ensure they met inclusion criteria. Guidelines were excluded that (1) were not the most recent version of the organization’s guideline; (2) targeted treatment for pediatric populations or were focused on gestational or type 1 diabetes; (3) were duplicate records. All guidelines meeting inclusion criteria were checked to ensure they were published in the USA and available in English. Titles and abstracts were then sorted using the reference management software EndNote version X9. The medical librarian supported the team to obtain all eligible full text guidelines.

### Data Synthesis and Analysis

The full text of each included guideline was scanned for references to social care activities using a keyword search, which included 37 social risk-related terms identified in prior research linked to social determinants of health [Appendix**]** [15, 16]. The social risk terms were categorized along 7 domains:

1. Financial security: recommendations related to decreasing economic hardship and barriers (keyword examples: poverty, income, bill)
2. Transportation: recommendations related to ensuring consistent and reliable means of transportation (keyword examples: accessibility, walk, walkability)
3. Access to health care: recommendations related to addressing barriers to quality health care such as lacking insurance, identifying a clinic. (keyword examples: coverage, primary care, resources)
4. Language and literacy: recommendations related to addressing health literacy level and preferred language (keyword examples: literacy, language, education)
5. Food security: recommendations related to improving sufficient, affordable, and nutritious foods (keyword examples: food, hunger, insecure)
6. Housing security: recommendations related to addressing challenges attaining affordable housing (keyword examples: homelessness, housing, home)
7. Sociodemographics: recommendations based on patient race/ethnicity, educational attainment, gender, and religion (keyword examples: culture, religion, race)

The keyword search identified relevant guideline passages related to social care. Any passage containing a relevant keyword subsequently was coded as relating to one or more (not mutually exclusive) of the five social care categories from the NASEM framework on social care activities as defined in the 2019 report [12].

Using NVivo 12, three team members (BA, MP, RG) initially engaged in a group process to abstract and code identified passages referencing social risks in 10 randomly selected guidelines. Members of the research team coded the same 10 guidelines for both the referenced social risk domain (e.g., food, housing) and any recommended social care activities per the NASEM framework. Any discrepancies in coding were discussed among group members, and differences in the interpretations of code definitions were explored until an agreement was reached. Specifically, examples for each code were provided in the codebook to inform coding decisions. This process helped the group establish consensus on code definitions. When coders did not reach consensus on any guideline text during this process, the three coders discussed as a group to reach agreement on the most appropriate code. All remaining guidelines were independently coded by one of the three coders, and team members met intermittently to discuss any questions or ambiguities. Queries were conducted to calculate the number of references in each social domain category and the number of social care recommendations in each of the NASEM social care categories. These data were extracted to produce a table that reflected the abridged citation, recommended social care activity, and social risk domain, which the research team reviewed for accuracy.

## Results

**Fig. 1:**
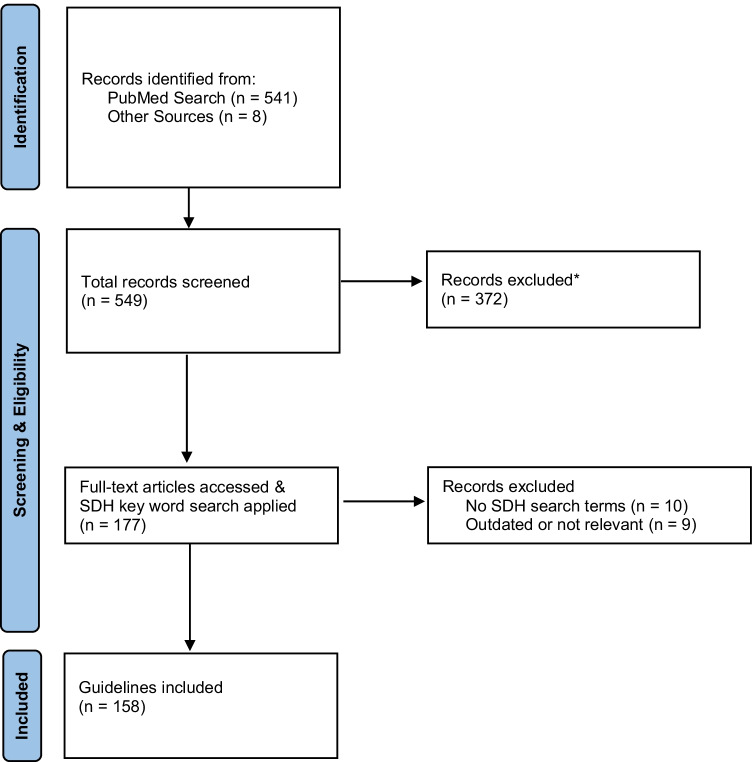
PRISMA Flow Diagram. * Reasons for exclusion: focus on select populations (pediatric) or conditions (pregnancy/gestational or type 1 diabetes); duplicate records; not a guideline)

One hundred and fifty-eight national T2D treatment and care guidelines met study inclusion criteria; 154 came from the initial PubMed search, and 4 were added from the subsequent search of professional T2D and endocrine association websites (see Fig. 1). The majority (122; 77%) of these guidelines did not include recommendations related to any of the five NASEM social care activities; the remaining 36 (23%) referred to one or more social care activities. Among the 36 guidelines that did refer to social care activities, the frequency of recommendations varied by social care category. While less than half of the guidelines (15/36; 42%) recommended *Awareness* activities and almost all recommended *Adjustment* activities (34/36; 94%), only eight (8/36; 22%) included recommendations related to *Assistance* activities; four guidelines (4/36; 11%) recommended *Alignment* activities, and five (5/36; 14%) recommended *Advocacy* activities. As shown in Table 2, more recently published guidelines are more likely to describe social care activities: three guidelines published before 2000 included at least one type of social care recommendations, while in 2022 alone, all guidelines included multiple types of social care recommendation. Four additional guidelines acknowledged the influence of social conditions on T2D outcomes in the absence of specific social care activity recommendations [17–20].

**Table 2:** Guidelines with social care activity recommendations and related social domains

Year	Authors	Number social care activity recommendations (social domains)				
		Awareness	Adjustment	Assistance	Alignment	Advocacy
1991	Howe RS, Christman C [37]		1 (Transportation) 1 (Sociodemographic)			
1994	Williams A [38]		1 (Financial security)			
1995	American Association of Diabetes Educators [39]		1 (Language and literacy) 1 (Sociodemographic)			
1995	Monk A, Barry B, McClain K, et al. [21]	2 (Financial security) 1 (Food security) 1 (Housing security) 2 (Sociodemographic)	1 (Language and literacy)	1 (Financial security) 1 (Sociodemographic)		
1997	American Association of Diabetes Educators [40]		1 (Financial security)			
2000	American Association of Clinical Endocrinologists [22]	2 (Financial security) 1 (Sociodemographic)	1 (Financial security) 1 (Sociodemographic)			
2003	American Dietetic Association [41]		2 (Financial security)			
2003	California Healthcare Foundation, American Geriatrics Society [23]		1 (Financial security) 2 (Language and literacy) 3 (Sociodemographic)			
2004	American Diabetes Association [42]		2 (Financial security) 1 (Sociodemographic)			
2004	American Telemedicine Association et al. [61]		1 (Access to health care)			
2006	American Society of Pain Educators [43]		1 (Financial security)			
2006	Mensing C, Boucher J, Cypress M, et al. [24]	1 (Financial security) 1 (Sociodemographic)	1 (Financial security) 1 (Language and literacy) 2 (Sociodemographic)		1 (Sociodemographic)	
2007	American Association of Clinical Endocrinologists [44]		2 (Financial security)			
2009	Marwick TH, Horden MD, Miller T, et al. [45]		3 (Financial security) 2 (Sociodemographic) 1 (Transportation)	1 (Sociodemographic)		
2011	Klonoff DC, Buckingham B, Christiansen JS, et al. [25]	1 (Financial security)				
2013	American Association of Clinical Endocrinologists [46]		1 (Financial security)			
2013	American Geriatrics Society [26]	1 (Financial security) 1 (Sociodemographic)	1 (Financial security) 2 (Sociodemographic)			
2014	Sunni M, Brunzell C, Nathan B, et al. [47]		12 (Sociodemographic)	1 (Sociodemographic)		
2016	Evert AB, Bode BW, Buckingham BA, et al. [27]	1 (Financial security)	2 (Financial security)			
2016	Hingonrani A, LaMuraglia GM, Henke P, et al. [48]		1 (Financial security) 1(Language and literacy)			
2016	Peter AL, Ahmann AJ, Battelino T, et al. [28]	1 (Access to health care)	1 (Access to health care)			
2017	Qaseem A, Barry MJ, Humphrey LL, et al. [49]		1 (Financial security)			
2018	Cefalu WT, Dawes DE, Gavlak G, et al. [50]		3 (Financial security)			2 (Access to health care) 2 (Financial security)
2018	Davidson P, Ross T, Castor C [29]	1 (Financial security) 3 (Food security) 1 (Language and literacy) 3 (Sociodemographic)	3 (Financial security) 3 (Food security) 3 (Sociodemographic)	1 (Access to health care) 1 (Food security) 1 (Transportation)	1 (Food security)	1 (Food security)
2018	Davies MJ, D’Alessio DA, Fradkin J, et al. [51]		6 (Financial security) 1 (Language and literacy) 1 (Sociodemographic)			1 (Financial security)
2018	Hafida S, Ganda OP, Gabbay RA, et al. [30]	1 (Access to health care) 1 (Language and literacy) 1 (Sociodemographic)	1 (Access to health care) 2 (Language and literacy) 2 (Sociodemographic)			
2018	Munshi M, Blair E, Ganda OP, et al. [31]	1 (Financial security) 1 (Food security)	1 (Financial security)	2 (Financial security) 2 (Food security) 1 (Transportation)		
2019	American Academy of Opthalmology [52]		2 (Financial security)	1 (Access to health care)		
2019	LeRoith D, Biessels GJ, Braithwaite SS, et al. [53]		1 (Access to health care) 1 (Financial security)			
2020	Powers MA, Bardsley JK, Cypress M, et al. [32]	4 (Financial security) 2 (Food security) 2 (Language and literacy) 5 (Sociodemographic)	2 (Access to health care) 5 (Financial security) 3 (Food security) 3 (Language and literacy) 8 (Sociodemographic) 1 (Transportation)	1 (Access to health care) 2 (Financial security) 1 (Food security) 1 (Language and literacy) 4 (Sociodemographic)	1 (Housing security) 3 (Sociodemographic)	2 (Sociodemographic)
2021	Grunberger G, Sherr J, Allende M, et al. [54]		1 (Financial security) 2 (Access to health care)			
2022	American Diabetes Association [33•]	3 (Food security) 3 (Housing security) 3 (Financial security) 1 (Access to Health Care) 1 (Language and literacy) 1 (Transportation) 2 (Sociodemographic)	1 (Food security) 1 (Housing security) 4 (Financial security) 2 (Access to health care) 2 (Language and literacy) 1 (Transportation) 3 (Sociodemographic)	2 (Food Security) 2 (Housing Security) 3 (Financial security) 2 (Access to health care) 2 (Language and literacy) 1 (Transportation) 1 (Sociodemographic)	1 (Food security)	
2022	American Diabetes Association [34]	1 (Language and literacy) 3 (Sociodemographic)	1 (Financial Security) 1 (Language and literacy) 1 (Sociodemographic)			
2022	American Diabetes Association [35]	1 (Sociodemographic)	1 (Language and literacy) 1 (Food security)			
2022	American Diabetes Association [36]	2 (Food security) 1 (Financial security) 1 (Access to health care) 2 (Sociodemographic)				
2022	American Diabetes Association^62^		1 (Financial security) 1 (Housing security)			

In the 15 guidelines that suggested clinical teams collect social risk/asset information from patients (Awareness activities), the predominant focus was on three social domains: food insecurity, financial insecurity, and language and literacy [21–32, 33•, 34–36]. Several guidelines also recommended collecting information on challenges to accessing health care and other sociodemographic factors that influence T2D treatment and management [21, 22, 24, 26, 28–30, 32, 33•, 34–36]. None of the 15 guidelines that mentioned awareness activities described specific tools or implementation strategies for social risk or asset screening. For instance, one guideline called on providers to assess patient barriers related to both income and literacy recommendations but did not include any recommendations regarding specific screening measures, workflows, screening frequency, or data documentation [33•].

*Adjustment* recommendations (changes to health care delivery based on patients’ social conditions) appeared in 34 guidelines. Recommendations again clustered around three social domains, in this case financial security, language and literacy, and other sociodemographic factors [21–24, 26, 27, 29–32, 33•, 34–54]. Financial resource strain-related adjustments included reducing medication expenses by changing type and/or number of medications [44, 49, 50]; changing dietary recommendations to limit food costs [29]; and reducing health care referrals and in-person visits to reduce transportation needs or visit co-pays [30, 45]. Adjustments to reduce language and literacy barriers to care included efforts to match patients’ health care services with language preferences and literacy level [23, 33•, 51]. Adjustments related to patients’ cultural backgrounds and religious traditions included recommendations to change nutrition and medications to accommodate religious holidays or events, e.g., reducing insulin doses by 10% to 30% for patients fasting during Ramadan [32, 34, 47].

*Assistance* recommendations (defined by NASEM as suggestions for health care teams to intervene directly on social barriers that might impede T2D management) appeared in 8 guidelines. These recommendations included referring patients experiencing social barriers to health care, government, and/or community-based services intended to reduce those barriers, e.g., referrals for patients experiencing food insecurity to food assistance programs [29, 31, 32, 33•, 40, 45, 47]. Other recommendations included helping to reduce barriers to accessing health care services [29, 32, 33•, 52].

References to *Alignment* activities appeared in four guidelines, each example encouraging health care delivery organizations to align internal practices and external investments to complement community socioeconomic development [24, 29, 32, 33•]. These included recommendations to support the design of healthy food delivery systems [29] and encourage consumer advisory boards to ensure community needs are reflected in organizational decisions related to T2D prevention, management, and education [24].

Recommendations in five guidelines reflected the NASEM framework’s social care *Advocacy* category, which encourages health care organizations to promote policies and resources that increase the availability of social care resources at the community level [25, 29, 32, 50, 51]. These recommendations included organizational advocacy to improve insulin affordability [50] and the availability of diabetes management resources [25, 51]. Another recommendation encouraged policy-oriented advocacy around improving neighborhood-level social conditions to better support healthy lifestyles as a means of preventing and treating T2D.[32].

## Discussion

In this review of 158 T2D treatment and management guidelines, 122 guidelines (77%) did not refer to social determinants of T2D or recommend social care-related activities, e.g., screening for social risk or intervening to reduce or mitigate social risk. While the 2016 guideline dedicated specifically to psychosocial care for persons with T2D from the ADA acknowledges the role of social conditions in shaping T2D management and treatment, it does not describe any concrete social care recommendations [55]. Though references to social determinants increase in more recent publications, the finding that the majority of these documents do not refer to social determinants of T2D and that so few outline care recommendations are striking in light of consistent and compelling evidence on the impact of social adversity on T2D outcomes and comorbidities [6•, 7].

Among the guidelines that do include specific patient-level social care intervention strategies, few suggest systematically identifying social risks as part of T2D care. For instance, in the 34 guidelines that make recommendations to adjust treatment based on social risk information, under half (15) recommend routinely screening for any social risk factors to inform those types of adjustments. Of the 8 guidelines that recommend providing some form of assistance related to social needs, just seven suggest some form of social risk assessment. In the same ADA report described above that is specifically dedicated to psychosocial care [55], no socioeconomic risk screening measure is recommended, though 28 other measures are described that assess other psychosocial constructs in clinical settings. Yet, here are several screening tools that have been widely implemented in community health centers and other health systems to assess several social risks, including the Protocol for Responding to Assessing Patients’ Assets, Risks, and Experiences (PRAPARE) Screening Tool and Centers for Medicare and Medicaid Services-Accountable Health Communities Health-Related Social Needs Screening Tool [56, 57]. This disconnect between social risk assessment and related patient-level interventions points to gaps in the literature on social risk screening—including on its benefits, unintended consequences, and implementation—which might explain why screening is not consistently included in the guidelines alongside other specific person-reported measures (e.g., depression or diabetes distress screening). More worrisome, however, is that recommending interventions without some form of systematic strategy for identifying patients who might benefit from these interventions might contribute to inequitable intervention implementation and impact.

More generally, the relative sparsity of references to social determinants and recommendations about social care in T2D treatment and management guidelines may reflect gaps in high-quality evidence demonstrating that specific social care interventions are effective for improving T2D outcomes. Yet a growing crop of research studies suggests that select patient-directed social care interventions can affect health outcomes—including outcomes specific to T2D [58, 59]. These types of social care studies will need to be carefully monitored over time by guideline developers to assess whether sufficient evidence has accumulated to warrant updating future guideline iterations.

Finally, the fact that 12 clinical treatment and management guidelines do include alignment and/or advocacy recommendations highlights a growing recognition that effective prevention and treatment of chronic diseases like T2D will likely require community-level interventions. The small total number of recommendations made on this topic; however, indicates that the role of the health care delivery system in those activities has yet to be firmly established.

### Limitations

Findings from this review should be interpreted in light of multiple limitations. First, only guidelines sponsored by professional health care organizations were included, though there are other evidence-based resources on T2D treatment and management that influence care for patients with T2D, including influential original research, scientific reviews, and national organization websites. Second, the research team limited this analysis to guidelines written in English; guidelines designed specifically for populations who prefer other languages, such as Spanish-speaking Latino populations, may be more likely to include additional social care-oriented activities. Third, in some cases, the guidelines did not provide sufficient details to categorize the recommendation. Using an iterative three-reviewer process, coders discussed recommendations that were difficult to code using the NASEM framework and reached consensus on best-fit categories for the majority of codes. Finally, the NASEM framework guided this review because it covers a wide range of activities (from patient-centered to community-focused), which enabled the research team to abstract more references to social care than would be possible using a different social care framework [60].

### Conclusion and Future Implications

Health care systems in the USA are increasingly turning to social care as a strategy for improving health and reducing health disparities. The global tragedy of the COVID-19 pandemic has underscored the need for this approach. In this study, we found that the majority of national T2D treatment and management guidelines do not recommend social risk assessment or social care interventions as a routine part of care for people living with T2D. In guidelines that do contain social care recommendations, there is little consensus on how to identify patients with social risks or on actions to reduce the impacts of social factors on T2D outcomes. In the future, multidisciplinary teams of guideline developers should intentionally seek out and review social care integration research as one potential component of efforts to reduce pervasive T2D health inequities.

## Appendix

Social risk keyword search terms

**Table:**

Financial security and access to health care	Transportation	Language and literacy	Food security	Housing security	Sociodemographic factors
Cost Poverty Poor Financial Affordability Discount Income Employment Debt Bill Insurance Economic Expense	Transportation Accessibility Access Walk Walkability	Education Literacy Language Training	Insecure Food Hunger	Home Homeless Homelessness Housing Neighborhood	Social determinants Resource constrained Resources Disparities Socioeconomic Socio-economic Sociodemographic Primary Care Coverage Health Coverage Culture Cultural Cultural competency Safety Violence Discrimination Bias Racism Race Racial Trust Distrust
